# Evidence for selection events during domestication by extensive mitochondrial genome analysis between *japonica* and *indica* in cultivated rice

**DOI:** 10.1038/s41598-019-47318-x

**Published:** 2019-07-26

**Authors:** Lin Cheng, Kyu-Won Kim, Yong-Jin Park

**Affiliations:** 1 Department of Plant Resources, College of Industrial Science, Kongju National University, Yesan, 32439 Republic of Korea; 2 Center for Crop Breeding on Omics and Artificial Intelligence, Kongju National University, Yesan, 32439 Republic of Korea

**Keywords:** Evolutionary genetics, Agricultural genetics

## Abstract

The history of the domestication of rice is controversial, as it remains unknown whether domestication processes occurred once or multiple times. To date, genetic architecture and phylogenetic studies based on the rice nuclear genome have been extensively studied, but the results are quite different. Here, we found interesting results for different selections in *Oryza sativa* based on comprehensive studies of the rice mitochondrial (mt) genome. In detail, 412 rice germplasms were collected from around the world for variant architecture studies. A total of 10632 variants were detected in the mt genome, including 7277 SNPs and 3355 InDels. Selection signal (*π*_*w*_/*π*_*c*_) indicated that the selection sites in *Oryza sativa* L. ssp. *japonica* were different from those of *Oryza sativa* L. *indica* rice. The fixation index (*F*_ST_) was higher between *indica* and *japonica* than between *indica* and wild rice. Moreover, haplotype and phylogenetic analyses also revealed *indica* clusters and *japonica* clusters that were well separated from wild rice. As mentioned above, our studies indicate that the selection sites of the *indica* type were different from those of the *japonica* type. This means that *indica* and *japonica* have experienced different domestication processes. We also found that *japonica* may have experienced a bottleneck event during domestication.

## Introduction

The domestication of rice is the process of transforming the natural selection process of wild characteristics into the stable desired traits from selection. For African rice, it is well established that *Oryza glaberrima* was independently domesticated from the wild rice *Oryza barthii*^1^. For Asian rice (*Oryza sativa* L.), although *Oryza rufipogon* is widely considered to be the ancestor of Asian rice, there is still controversy about the occurrence of single or multiple domestication processes^2,3^. Asian rice is mainly divided into two major varieties, namely, *indica* and *japonica*. Generally, the *indica* type usually shows thin and long grain and is planted in tropical Asia; *japonica* rice usually shows short and sticky grain and is planted at high altitudes in South Asia. Both *indica* and *japonica* are important food crops for nearly half of the global population^4^. Exploring the genetic information of these diverse varieties can provide deep insights into rice domestication and breeding.

One of the most basic and controversial issues regarding Asian rice is the number of times domestication occurred^2^. Traditionally, molecular markers (microsatellites) were used to study certain domesticated genes for domestication history^5,6^. Since these molecular markers represent some part of the rice genome, whole-genome sequences of rice were used to improve this situation^7^. However, for nuclear genomic studies of rice, the results are completely different due to introgression, bottleneck events or materials^8,9^. Generally, for single-domestication studies of rice, the ‘domesticated loci’ that exist in both *indica* and *japonica* provide strong evidence to support the single domestication of rice^10,11^. The large genomic differences and breeding barriers present in *indica* and *japonica* directly support multiple independent domestications^9,12^. Due to the influence of gene flow and bottleneck events, some hypotheses have emerged, such as a single domestication with multiple origins or single origin with multiple introgressions^11,13,14^. As mentioned above, the history of Asian rice, specifically whether Asian rice stems from a single domestication event or from multiple domestications, remains unknown. Therefore, exploring the genetic information of *Oryza sativa* is very important as such information may provide more evidence for the domestication of rice and many important insights into the breeding of elite varieties for sustainable agriculture.

Mitochondria are important plastids that provide energy for the growth and evolution of plants. For the mt genome, the genome size ranges from ~200 kb to 2 Mb mostly, and mitochondria have specific modes of gene expression in higher plants^15^. Since the first entire sequence of the rice mitochondrial genome (490,520 bp) was reported in 2002^16^, mt analysis has been a powerful tool for us to understand the evolutionary history of rice due to the apparent lack of recombination, maternal inheritance, high copy number, and substitution rate^17–20^. Although there are some detailed explorations about rice mitochondrial genome such as the variations of rice mitochondrial genome and the comparison between nuclear and chloroplast genome, it is rare to associate them with the domestication of rice^19–21^. What’s more, most of these studies are limited to certain genes or to certain locations of the mitochondrial genome and do not provide evidence for comprehensive analysis. Therefore, we have used 412 rice varieties aiming to provide a comprehensive analysis of the mitochondrial genome to deepen our understanding of the rice genetic and evolutionary background.

Here, we conducted genetic variant analyses of 412 rice germplasms to investigate the evolutionary history of *Oryza sativa*. First, 358 Asian cultivated rice and 54 wild rice samples were collected from around the world to detect single nucleotide variants (SNPs), and insertions and deletions (InDels) based on the rice mitochondrial genome of *Oryza japonica*. Then, we used the selective sweep, *F*_ST_, haplotype network and phylogenetic tree to comprehensively mine the genetic background of Asian rice. Our analysis focuses on the genetic architecture of the rice mt genome, which provides more insight into the evolutionary history of *Oryza sativa*.

## Results

### Variants in the mitochondrial genome

The accession information and genome sequencing of all samples are summarized in Supplementary Table S1. A total of 412 rice samples were collected from various parts of the world and sequenced with high average coverage (~16X), yielding ~3.42 TB of read data. The entire collection included 253 *temperate japonica*, 25 *tropical japonica*, 66 *indica*, 9 *aus*, 2 *aromatic* rice, 54 wild rice, and 3 admixture types. These germplasms were aligned to the reference mt genome of *Oryza sativa japonica* [NC_011033.1] for variant calling.

**Figure 1:**
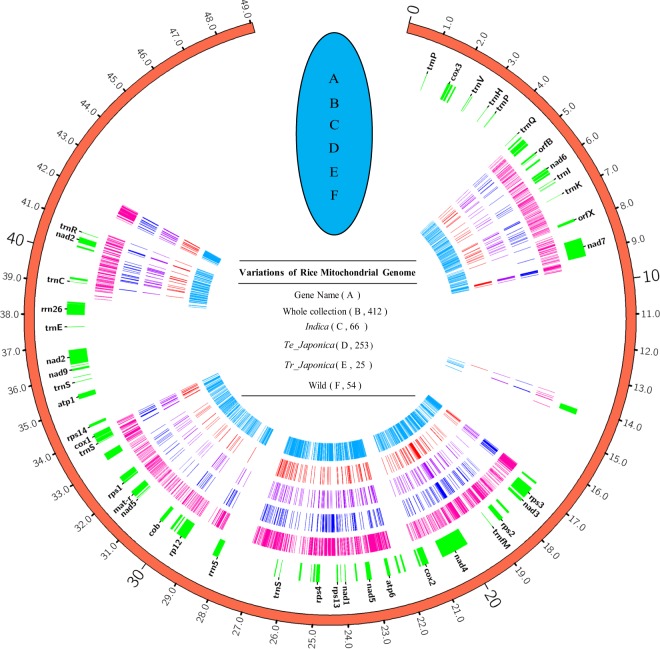
The band distribution of variants (SNPs and InDels) across the mitochondrial genome. The band position is depicted as the distance of the first variant of SNPs or InDels based on the reference genome of *Nipponbare*. (**A**–**F**) Highlights marked on the circle map indicate the SNP and InDel positions. (**A**) The label name of each gene located in the mitochondrial genome based on the position of the reference genome. (**B**) Total variants detected among the 412 accessions. (**C**) Variants identified in the *indica* subgroup. (**D**) Variants identified in the *temperate japonica* type. (**E**) Variants identified in the *tropical japonica* type. (**F**) Variants in wild rice. The outside distance unit is kb. The number inside the brackets indicates the number of each accession. On account of space, not all genes are illustrated in the figure.

A total of 10,632 primary variants were identified from the rice mt genome, including 7,277 SNPs (68.4%) and 3,355 InDels (31.6%) (Table 1). Since the number of each subgroup is different, we also summarized the average of the variants for each sample (Supplementary Table S2). For all SNPs, transitions appeared most frequently, accounting for 65.3% of all SNPs, almost 2 times of transversions. The type of variant is also summarized, revealing that G/A and C/T seem to be more likely to appear in the mt genome, followed by A/T and T/C (Supplementary Fig. S1 and Supplementary Table S1). After filtering minor allele frequencies (MAFs) <0.01 and variants >20% missing calls, 2,159 high-quality (HQ) variants were obtained for subsequent statistical analysis^22,23^. For Asian rice, we detected a total of 755 HQ variants, with 75 HQ variants (9.9%) located in the open read frame (ORF) and 52 HQ variants (6.8%) located in the coding region. Among *Oryza sativa*, we found 49 common SNPs that appeared in 5 subgroups, which means that these SNPs are almost fixed in rice and may play important roles in the mitochondrial genome (Supplementary Fig. S2a). Furthermore, we detected 48 of the same SNPs in *O. rufipogon* and *O. nivara* compared with the 49 same SNPs in *Oryza* sativa (Supplementary Fig. S2b). This means that these common variants (48/49) of *Oryza sativa* may come from wild rice, and one mutation (1/49) appeared and became fixed due to the drive of selection during domestication. The variants’ distribution in the whole accession and different groups were also targeted based on the reference genome, revealing that wild rice has the highest variant, followed by *indica* (Fig. 1). Interestingly, these variants showed a cluster distribution in each subgroup, indicating that certain positions of the mitochondria are not allowed to change. This is consistent with a highly conserved mitochondrial genome.

**Table 1:** Summary of the total and subgroup variants (SNPs and InDels) detected in 358 cultivated rice along with 54 wild rice samples collected from different countries around the world.

Summary	Type	Mt^a^ Variant					Mt^a^ HQ^b^ Variant			
	SNPs	7,277					1,764			
	InDels	3,355					395			
	Total	10,632					2,159			
	Type	No. of Accession	Variant				HQ^b^ Variant			
			SNPs	InDels	Total	Ts/Tv	SNPs	InDels	Total	Ts/Tv
Subgroup	Cultivated	358	1,437	508	1,945	1.956	646	109	755	2.091
	Wild	54	6,746	3,122	9,868	1.884	1,625	348	1,973	1.559
	*Indica*	66	1,000	383	1,383	1.985	545	99	644	2.187
	Te_*japonica*	253	908	266	1,174	1.759	430	51	481	2.028
	Tr_*japonica*	25	682	214	896	1.877	329	42	371	2.391
	*Aus*	9	549	202	751	1.553	351	71	422	1.949
	*Aromatic*	2	189	77	266	0.909	63	8	71	1.52
	Admixture	3	662	177	839	1.669	314	37	351	2.048

Ts/Tv is the proportion of transition/transversion. Te_*japonica*: *temperate japonica*; Tr_*japonica*: *tropical japonica*.

**Mt**^**a**^ **Variants:** All mitochondrial genome variants in our study.

HQ^b^ Variants: High-quality variants. Here, we removed 80% of missing data and minor allele frequency (MAF) < 0.01.

Mt^a^ HQ^b^ Variants: High-quality variants of the mitochondrial genomes in our study.

### The evidence of different selection in *Oryza sativa*

**Figure 2:**
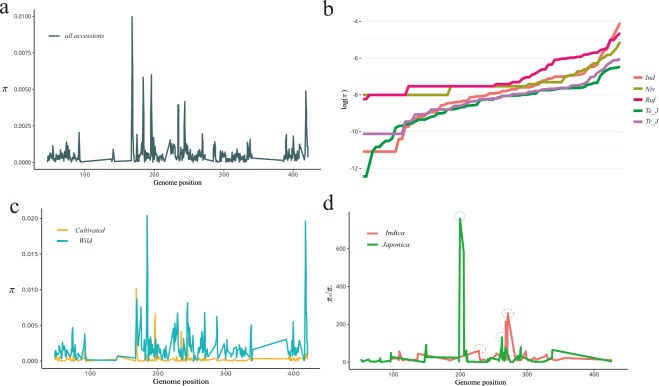
Nucleotide diversity and selection analysis of all accessions and subgroups. (**a**) Nucleotide diversity of all accessions. A 500 bp window size was used in this analysis. (**b**) Nucleotide diversity of subgroups. The sorted values were plotted in each group. *Ind*: *indica*; *Niv*: *O. nivara*; *Ruf*: *O. rufipogon*; *Te_J*; *temperate japonica*; *Tr_J*; *tropical japonica*. (**c**) Nucleotide diversity of cultivated rice and wild rice. (**d**) The reduction in nucleotides was calculated based on previous diversity analysis. The threshold of the top 5 percentile is indicated as a red dotted circle for *indica* and blue circle for *japonica*. The regions within the 2.5 percentile are considered candidate regions under selection. The genome position unit is kb.

In genetic analysis, different methods lead to different conclusions due to the presence of hybridization or introgression events^8,9^. Since mitochondria are highly conserved genomes of maternal inheritance, there is almost no genetic recombination through hybridization. Therefore, whether gene flow present between *indica* and *japonica* rice has an important impact on our subsequent analysis at the mt genome level. For recent gene flow, we analyzed the frequency of each group and their distribution based on a physical map of the reference genome^7^. In our results, we found that *indica-specific* sites (allele frequency >95% in *indica*) were different from *japonica* sites (allele frequency <5% in *japonica*), which means that there is no gene flow or introgression event in the rice mt genome (Supplementary Table S4). Therefore, our analysis of the genetic history of the rice mitochondrial genome is trustworthy. For rice domestication studies, we first examined the *dN/dS* ratio (*nonsynonymous substitution rate*/*synonymous substitution rate*) of Asian cultivated rice to calculate the evolution rate by coding region (Supplementary Fig. S3). A total of 75 genes were identified from all subgroups, and 23 genes exhibited positive selection. To identify specific positions of rice that were selected, we performed selective sweep analysis based on the diversity of rice in the mitochondrial genome. The diversity of the rice mitochondrial genome ranges from 3.7 × 10^−5^ to 2.0 × 10^−2^ (Fig. 1A) (Supplementary Table S5). Wild rice exhibited higher diversity than Asian cultivated rice (*P* < 0.01) (Fig. 2c). The diversity of subgroups was also analyzed based on the whole variations (SNPs and InDels), and *japonica* has a lower diversity compared to the other subgroups (Fig. 2b) (Supplementary Table S6). Based on the analysis of diversity, we used *π*_*wild*_*/π*_*cultivated*_ of the top 5% cutoff of each Asian rice to determine selection sites (Supplementary Table S7). For 5% cutoff values, we detected a total of 8 selection sites, 4 selection sites for the *indica* type, and 4 sites for the *japonica* type. If *indica* and *japonica* were only domesticated once, they should be roughly similar in selection sites. Here, in 4000 bp cutoff areas, we only detected a 500 bp (12.5%) similar area between *indica* and *japonica* type. The selective sweep of RAiSD analysis was also conducted, which used *μ* statistics to detect positive selection based on multiple signatures^24^ (SFS, LD, and diversity) and SNPs (Supplementary Fig. S4). The results revealed that one region of *japonica* (100–150 kb) had experienced strong selection compared with *indica*.

### *F*_ST_, Tajima’s D test, PCA and MDS of populations

**Figure 3:**
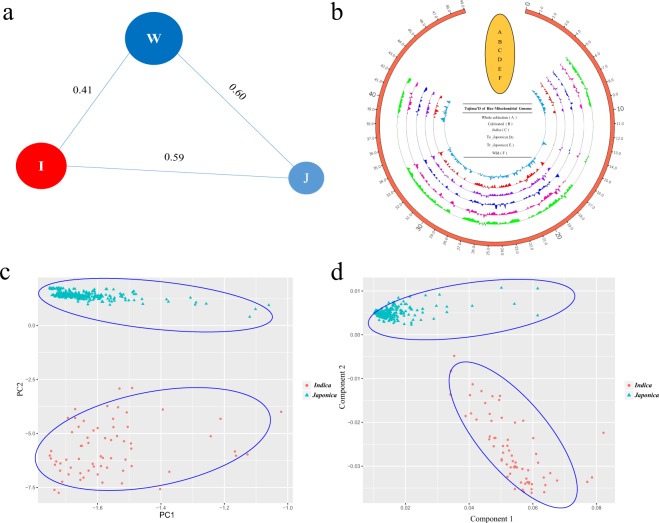
*F*_ST_, Tajima’s *D* test, principal component analysis and multidimensional scaling of populations. (**a**) The *F*_ST_ value between Asian rice and wild rice; the circle size displayed the diversity of each group. The *F*_ST_ value between each group was marked by the length of each line. R: *O. rufipogon*, Tr: *tropical japonica*, I: *indica*, Te: *temperate japonica*, N: *O. nivara*. (**b**) Tajima’s *D* values in subgroups based on the rice mitochondrial genome. (**c**) Principle component analysis of *indica* and *japonica*. (**d**) Multidimensional scaling plots of *indica* and *japonica*.

The fixation index (*F*_ST_) was used to determine the degree of differentiation in *Oryza sativa* based on weighted methods^25^. *Indica* and *japonica* displayed higher *F*_ST_ values compared with wild rice in the mt genome (Fig. 3a). This finding also indicates *indica* and *japonica* rice may have reproductive barriers, although the fertility of hybrids varies from individuals^26^. For Tajima’s *D* value, *temperate japonica* and *tropical japonica* had a similar curve, and *indica* was shown the different curve in some part of rice mt genom*e* compared with *japonica* (Fig. 3b). Principal component analysis (PCA) and multidimensional scaling (MDS) discriminated two statistically different groups of Asian cultivated rice (*indica* and *japonica*) (Fig. 3c,d). As described above, these findings indicated that mainly Asian rice *indica* and *japonica* may have far genetic distances and different genetic backgrounds.

### Haplotype network, population structure, and phylogenetic tree

**Figure 4:**
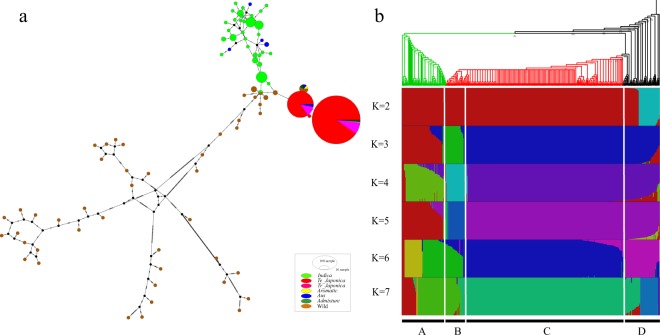
The haplotype network, population structure and phylogenetic tree of 412 rice accessions. (**a**) The haplotype network of 412 rice accessions. Here, different colors represent different populations, and circle size represents the number of samples. (**b**) Population structure and phylogenetic tree are displayed using a rectangular cladogram. A: *indica*; B: *tropical japonica*; C: *temperate japonica*; D: *aromatic*, *aus* and admixture type; E: wild rice.

A total of 85 haplotypes were detected from 412 rice samples by DnaSP v6 based on high-quality variations^27^. Among these haplotypes, 38 haplotypes and 47 haplotypes were found in Asian rice and wild rice, respectively. In Asian rice, *indica* exhibited 31 haplotypes, whereas *japonica* only exhibited 4 haplotypes. If *indica* and *japonica* were domesticated once, they would have very similar haplotypes. However, we did not identify any shared haplotypes between these two subgroups at the mt level (Fig. 4a). Moreover, population structure from K = 2 to K = 7 were used to entirely distinguish the individual subgroups among the entire collection. To more accurately determine the structure, K = 5 was estimated by ChooseK.py in fastStructure (Fig. 4b). For K = 5, although *japonica* and wild rice are mixed together, we found a clear separation of *indica* and *tropical japonica*. We also found that the same composition exists in *indica* and *japonica* (purple color), but this composition was also found in wild rice and does not provide evidence for *indica-specific* or *japonica-specific* structure. This same structure could be obtained independently from the wild rice during a separate domestication^28^. To accurately assess the domestication relationship, we used all HQ SNPs to construct a phylogenetic tree using the Bayesian inference method. If Asian rice was only domesticated once, a tree with these two subpopulations as mixed or sister taxa should be most strongly supported^29^. However, in our results, *japonica and indica* types were clearly separated from wild rice (Fig. 4b). The archaeological evidence of *Oryza sativa* (>9,000 years) in India and China^30,31^ also exhibited independent domestication of Asian rice. As described above, these results demonstrate that *indica* and *japonica* may have a distinct genetic background, which supports the concept of multiple independent domestications of Asian rice.

## Discussion

The domestication history of *Oryza sativa* is complex. Although numerous studies on the origin of Asian rice have been conducted, results regarding whether single or multiple domestications occurred are still controversial^32–35^. Hybridization and gene flow in the natural state are two important factors affecting rice origin studies^36–38^. Hybridization is the critical step that brings together the high-quality features of the parents, thus disrupting the unique components of different subspecies for evolutionary studies^39–41^. Gene flow is the selection of genes from one species and the movement of such genes to the gene pool of another species^13,42^. Gene flow results in the genetic differentiation of local populations and plays an important role in genetic studies of specific loci in subgroups^28^. Civáň *et al*. (2018) argued that there are some potential alleles that moved to other populations by introgression events in rice, which have a critical impact on distinguishing and understanding the real history of *Oryza sativa*^28^. Wang *et al*. (2017) demonstrated that the different conclusions from rice genome analyses are due to extensive, continuous gene flow from cultivated rice to wild rice^35^. Fortunately, the mt genome is maternally inherited, and almost no genetic recombination occurs in the natural state, which provides pure and trustworthy materials for phylogenetic studies^42,43^. We did not detect any introgression signals between *indica* and *japonica* at the mt level based on statistical analysis of allele frequency^7^. Therefore, our mt genome architecture with high-quality variants is useful for solving contradictions in the domestication of Asian rice.

In the evolutionary history of rice, Huang *et al*. (2016) argued that *japonica* experienced a strong bottleneck event and that the cutoff of *π*_*w*_*/π*_*c*_ should be accurate for collocated low-diversity genomic region (CLDGR) detection^13^. Based on this, we used the top 5 percentile of genetic diversity for a better selective sweep investigation. We detected a strong selection signal present in *japonica* rather than *indica*. In recent articles, the strong bottleneck effect was also revealed in *japonica* by genome-based position and the magnitude of selective sweeps^13,44,45^. Our selective sweep results of the rice mt genome were consistent with previous reports that performed chloroplast genome and nuclear analysis, which demonstrated that bottleneck event occurred in *japonica* during domestication. The comparison of the specific low diversity of a particular group may not necessarily mean independent domestication, as some selection pressures lead to areas of low diversity that may be adaptable to the local environment after the separation of Asian rice. However, if all cultivated rice came from a single domestication, the selective sweep during this event is expected to generate some of the same curves in subspecies. In our results, the selective sweep site in *japonica* was different from that of *indica*. Principal component analysis and population structure also confirmed this finding, showing distinct genetic information based on high-quality (HQ) SNPs. Furthermore, the phylogenetic analysis revealed 2 clusters of *indica* and *japonica* from wild rice. As described above, this means that *japonica* and *indica* may have been selected differently during domestication.

## Methods

### Samples and resequencing

A heuristic set containing 358 rice accessions with 3 types of accessions (landraces, weedy, and bred) previously generated from worldwide varieties collected from the National GeneBank of the Rural Development Administration (RDA-Genebank, Republic of Korea) using the program PowerCore^46^ was selected for whole-genome resequencing^47^. In addition, 54 wild rice accessions were obtained from the International Rice Research Institute (IRRI) in 2017.

For the 358 Asian rice and 54 wild rice accessions from our database, plants were planted in a soft field with enough water. After checking the heading date (approximately 13 days), young leaves were sampled from one plant and stored at –80 °C prior to genomic DNA extraction using the DNeasy Plant Mini Kit (Qiagen). Qualified DNA was used for whole-genome resequencing of the collected rice varieties with an average coverage of approximately 16X on the Illumina HiSeq. 2000 Sequencing Systems Platform.

### Variant calling and data management

The assembly process included data preparation, filtering, mapping, sorting, and variant calling. First, the index was processed by Burrows-Wheeler Alignment v 0.7.15 (BWA)^48^, Samtools v1.3.1^49^ and Picard v 2.14 (http://broadinstitute.github. io/picard/) before variant calling. Second, raw data were aligned to the *Nipponbare* mt genome sequence (https://www.ncbi.nlm.nih.gov/nuccore/NC_011033.1) using BWA. A sequence alignment map (SAM) file was created during mapping and converted to a binary SAM (BAM) file with sorting. Then, removal of duplicates and the addition of reading group IDs were performed using Picard Tools. Final realignment and identification of variants were performed using GATK v 3.7. Statistical analyses were applied to summarize the number and distribution of variants based on the Haplotype Map (HapMap) file generated from the VCF file. Default settings were used for most software and tools.

### Statistical analysis and PCA

Statistical analyses of nucleotide diversity (*π*) and the fixation index (*F*_ST_) were conducted using Vcftools v 0.1.15^50^ with a 1000-bp slide window and 500-bp steps for all collections and individuals. The *F*_ST_ value was used to determine the degree of population differentiation. The significance of diversity in the group was assessed using t-tests. For introgression event analysis, we followed Zhao’s method^51^. Generally, highly differentiated alleles of SNP loci were identified among *indica*, *temperate japonica*, and *tropical japonica*. SNP loci had an allele frequency greater than 0.95 in *temperate indica* and less than 0.05 in *indica*. At the SNP locus, allele information (*indica-specific* type, *temperate*-*indica-specific* type or *tropical-japonica-specific* type) of each accession was called across the mitochondrial genome. For each accession, the size of the introgression fragment in the genome was determined to estimate the proportion of potential introgression events. The selection effect of the geographic population was generated using Bottleneck v 1.2.02^52,53^ according to the allele frequency of each site. Regarding the reliability of the results for the detection of population bottleneck effects, minor allele frequencies <0.05 were removed from our data. To evaluate the relationship and population structure, PCA and MDS were conducted using TASSEL5 based on high quality SNPs to provide basic evidence of the population structure. Data were displayed with different groups and colors using the R package ggplot2 (https://cran.r-project.org/web/packages/ggplot2/index.html).

### Haplotype network and *dN*/*dS* ratios

The TCS^54^ haplotype network was generated using PopART v 1.7^55^. First, we used a python script to make FASTA data from the vcf file. Then, FASTA data alignment and transformation to nex format was performed using MEGA7. DnaSP v6^27^ was employed for haplotype analysis (Supplementary Table S9). For *dN*/*dS* analyses, all orthologous mt genes from 23 species were aligned to the paml format using prank^56^. Gblocks v 0.91b^57^ was applied to eliminate the conservation area of the ML tree (MEGA7). The maximum likelihood method of codeml of PAML v 4.9h^58^ was used to estimate the ω ratio with F3X4 codon frequencies. The branch test of the null hypothesis (model = 0, NSsites = 0) was used for a single ω across branches, and the model alternative hypothesis (model = 2, NSsites = 0) was used for ω per branch site. The likelihood ratio test (LRT) was used to identify accelerated genes in the rice group. Here, *indica* and *japonica* were assigned as foreground branches, and other accessions were assigned as background branches. Genes with ω > 5 were removed because they were considered outliers^59^.

### Population structure and evolution research

Briefly, fastStructure v 1.0^60^ was used to investigate population clusters. InDels were removed from all high-quality (HQ) variants to obtain SNP only vcf file. Given increased K values ranging from 2 to 7, the subpopulation of an individual ancestry could be completely investigated. Bayesian inference methods were applied to construct a phylogenetic tree for the 412 accessions based on the HQ variants. After removing missing data and gaps from whole positions, the phylogenetic tree of evolutionary history was conducted by MrBayes v3.2.7^61^ with the best nucleotide parameter (TVM + G) estimated by detection from 88 models with the software of JModelTest v 2.1.10^62^, with 1000 replicates and 6 categories.

## Supplementary information

Supplementary Materials

Dataset 1

## Data Availability

The datasets supporting the conclusions of this article are included within the article and its additional files. In addition, the raw VCF file generated from current 412 rice accessions were also deposited in the European Variant Archive Database under Project ID: PRJEB31784.
