# Supplementary material for: Evidence for selection events during domestication by extensive mitochondrial genome analysis between japonica and indica in cultivated rice
Source: Sci Rep. 2019 Jul 26;9:10846. doi: 10.1038/s41598-019-47318-x (PMC6659709; doi:10.1038/s41598-019-47318-x)
Supplement: Supplementary file 1 — Supplementary Materials [file 41598_2019_47318_MOESM1_ESM.pdf]

Supplementary Material for ‘Evidence for selection events during domestication by extensive mitochondrial genome analysis between *japonica* and *indica* in cultivated rice’

Lin Cheng[1], Kyu-Won Kim[2*]&Yong-Jin Park[1,2*]

[1]Department of Plant Resources, College of Industrial Science, Kongju National University, Yesan 32439, Republic of Korea

[2]Center for Crop Breeding on Omics and Artificial Intelligence, Kongju National University, Yesan 32439, Republic of Korea

[*]Correspondence: kyuwonkim@kongju.ac.kr (KK); yjpark@kongju.ac.kr (YP)

Supplementary information

Supplementary Figures

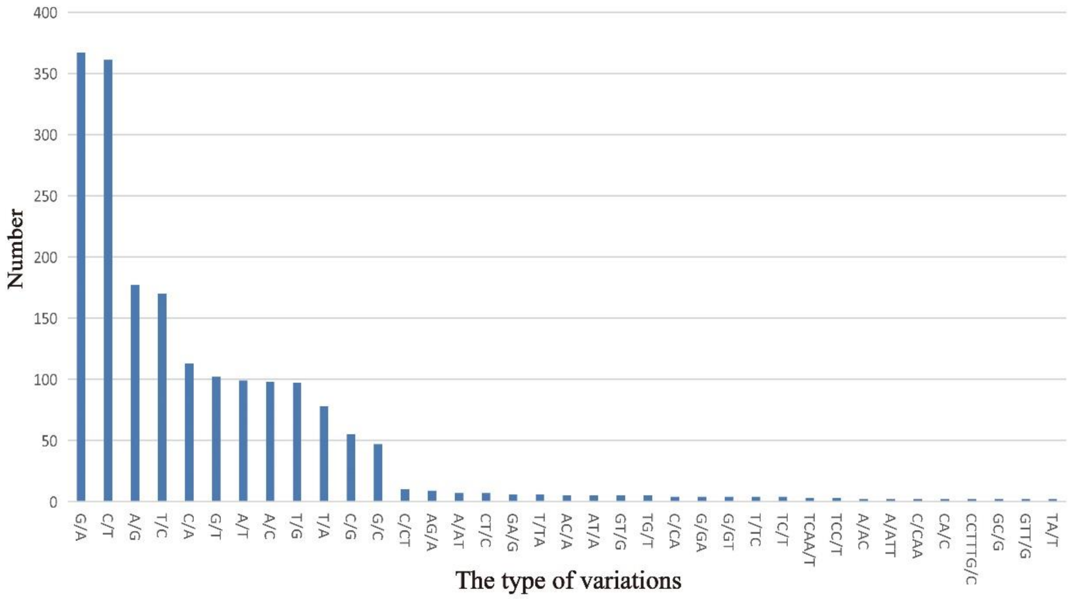

Supplementary Figure S1. The number of different types of variants. Here, the summary of the high-quality variants is shown. Note only these number of variants more than once are shown in this figure.

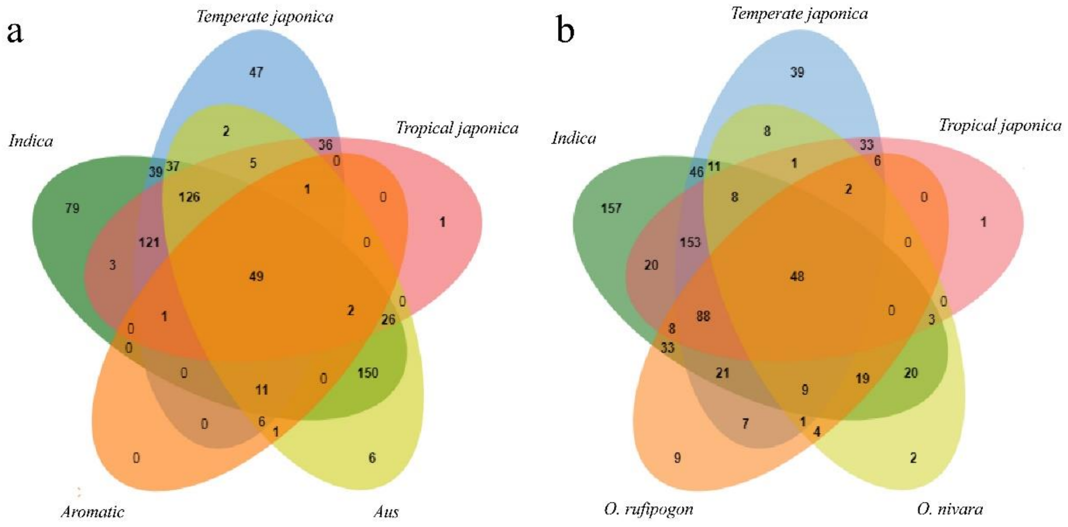

Supplementary Figure S2. The number of variants in subgroups. (a) Venn diagram of Asian rice (*temperate japonica*, *tropical japonica*, *aromatic*, *aus* and *indica*). (b) The number of variants in *japonica*, *indica*, *O. rufipogon*, and *O. nivara*. The number in the figure indicates the same SNV position in each population, and different colors represent different subgroups.

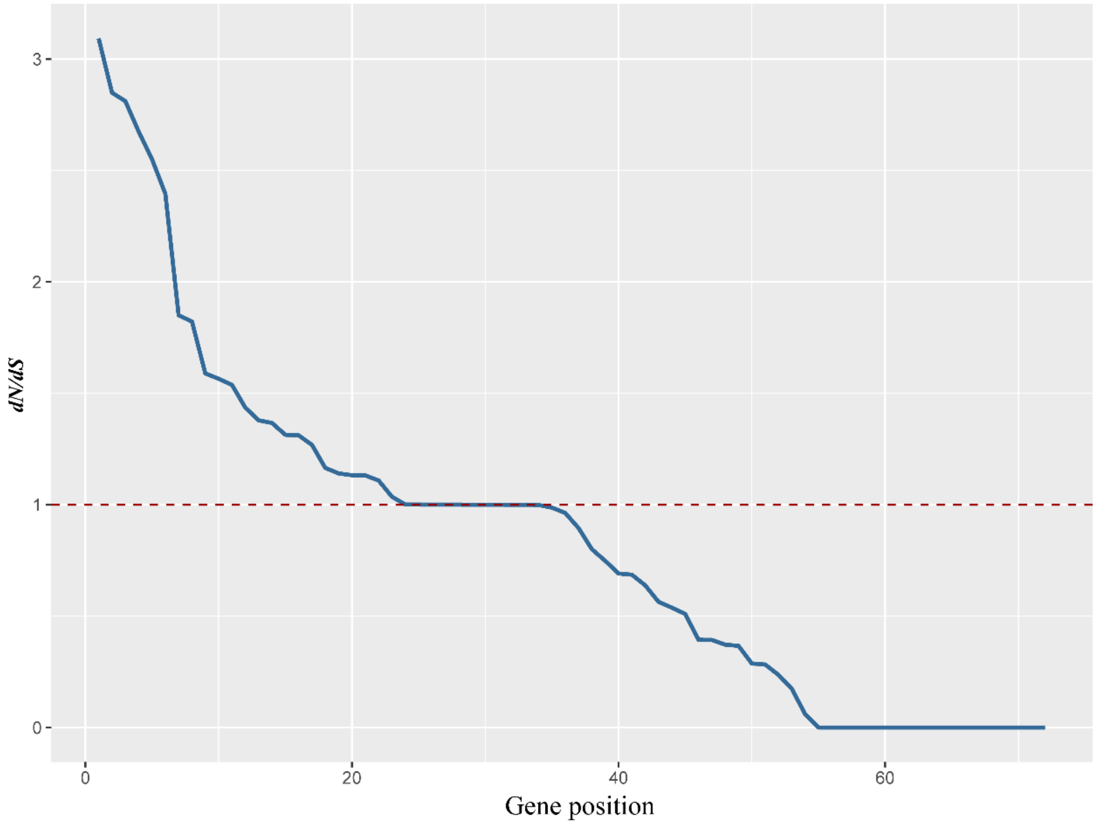

Supplementary Figure S3. *dN/dS v*alues of all genes in the mt genome. The decrease in Ka/Ks values of 87 selection genes in 23 typical rice accessions.

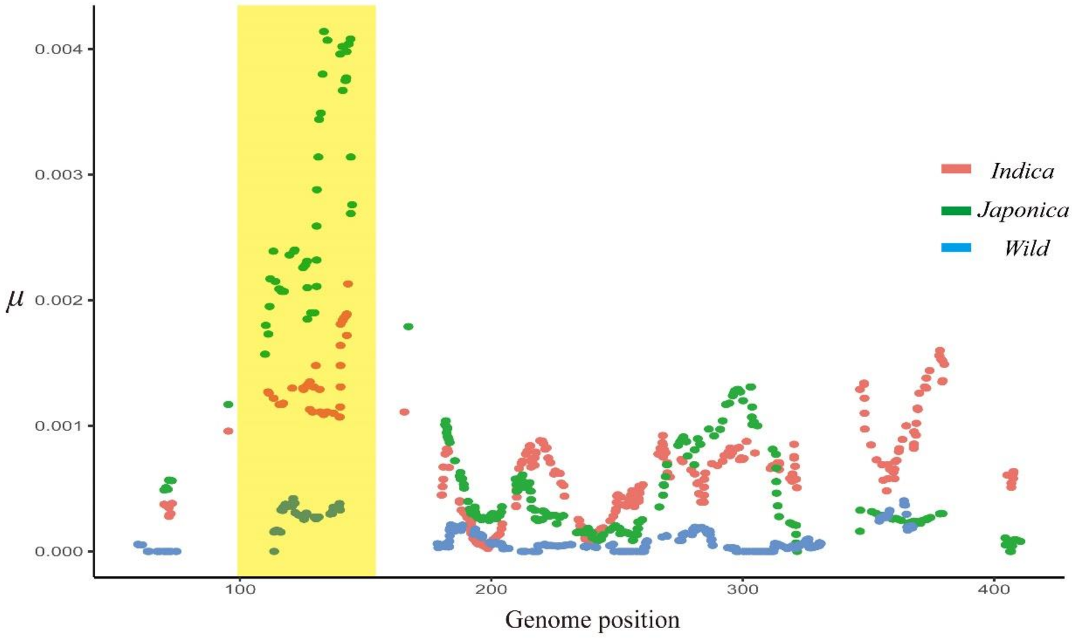

Supplementary Figure. S4. The selective sweep analysis of RAiSD in cultivated rice and wild rice. The x-axis represents the location of the genome and the y-axis represents μ values.

Supplementary Tables

Supplementary Table S1. Summary of the information of the total samples.

Supplementary Table S2. Summary of the total variants among the subgroups.

Supplementary Table S3. Location of all the SNPs and InDels in their coding region in the reference detected in this study.

Supplementary Table S4. Allele frequency information of *indica*, *temperate japonica* and *tropical japonica*.

Supplementary Table S5. Comparison of nucleotide diversity among all samples, Asian rice, and wild rice.

Supplementary Table S6. Comparison of nucleotide diversity of *indica*, *O. rufipogon*, *O. nivara*, and *japonica*.

Supplementary Table S7. Reduction in nucleotide diversity between indica and japonica in the cp genome.

Supplementary Table S8. Comparison of Tajima's *D* values of all accessions and subgroups.

Supplementary Table S9. The result of haplotype

Supplementary Table 1-9 are in separate files.
